# Screening of Antagonistic Bacteria against Three Aquatic Pathogens and Characterization of Lipopeptides in *Bacillus cereus* BA09

**DOI:** 10.4014/jmb.2404.04017

**Published:** 2024-08-25

**Authors:** Xinran Shi, Weijia Zhou, Xiaocen Lu, Cuiyan Cao, Dong Sheng, Xu Ren, Nanlin Jin, Yu Zhang, Zhixin Guo, Shengnan Cao, Shigen Ye

**Affiliations:** 1 Aquatic Animal Hospital of Dalian Ocean University, Dalian Ocean University, Dalian 116023, P.R. China; 2 Dalian Institute of Chemical Physics, Chinese Academy of Sciences, Key Laboratory of Separation Science for Analytical Chemistry, Dalian 116023, P.R. China

**Keywords:** *Bacillus cereus*, antibacterial, aquatic pathogens, 2D-LC-MS/MS, lipopeptides

## Abstract

Screening for antagonistic bacteria on aquatic pathogens and identification of antagonistic ingredients are essential to reduce the use of chemicals in aquaculture. In this study, strain BA09, subsequently identified as *Bacillus cereus*, simultaneously displayed strong antagonistic effects on *Edwardsiella tarda*, *Vibrio harveyi*, and *Streptococcus anisopliae* in the initial screening and rescreening. In addition, the methanol extract of BA09 was subjected to antibacterial activity verification and one-dimensional (1D) reversed-phase liquid chromatography (RPLC) preparation. A total of 27 fractions were collected, 6 of which were subjected to two-dimensional (2D) RPLC separation and tracked as antibacterial. A total of 14 lipopeptides that included 9 fengycin homologs, 3 bacillomycin homologs, and 2 surfactin homologs were identified by tandem high-resolution mass spectrometry. Through characterization of the antibacterial substance in *Bacillus cereus* BA09, which simultaneously inhibited *E. tarda*, *V. harveyi*, and *S. agalactiae*, the current study provides a theoretical basis for the development of antibacterial drugs in aquaculture.

## Introduction

Aquatic pathogens not only jeopardize the growth and development of cultured species but also seriously threaten the development of the aquaculture economy. *Edwardsiella tarda*, *Vibrio harveyi* and *Streptococcus agalactiae*, as three common aquatic pathogens, are widely distributed and highly pathogenic, while also showing an expanding host range. For example, *E. tarda* has been reported to infect Senegalese sole (*Solea senegalensis*) [1], striped catfish (*Pangasianodon hypophthalmus*) [2] and turbot (*Scophthalmus maximus*) [3]. Diseased fish typically exhibit skin lesions and hemorrhage in various parts of the body. The species infected by *V. harveyi* include Asian sea bass (*Lates calcarifer*) [4], Arabian surgeonfish (*Acanthurus sohal*) [5], and shrimp (*Penaeus monodon*) [6], with hemorrhagic skin ulcers being the primary clinical signs. *S. agalactiae* has also caused morbidity in certain species, such as hybrid tilapia (*Oreochromis niloticus*) [7] and giant Queensland grouper (*Epinephelus lanceolatus*) [8], with common clinical signs including erratic swimming, exophthalmia, and surface hemorrhages. Antibiotics are commonly used in the treatment of diseases caused by aquatic pathogens, but the hidden dangers are increasingly recognized. These include antimicrobial resistance, environmental pollution, and drug residues. The abovementioned three pathogens not only have a unique drug resistance spectrum but also show gradually increasing range and multi-drug resistance [1, 9, 10]. Thus, finding green and harmless prevention and control measures is urgently needed. Biological control technology using microecological agents is a current research hotspot due to its environmental friendliness and ability to improve immune performance while promoting the growth of cultured varieties [11].

Currently, a variety of bacteria have been reported to act as microecological agents through microbial antagonism. Regarding the antagonism of the abovementioned three aquatic pathogens, *Bacillus velezensis* LF01 has a strong antagonistic effect on *E. tarda*, *V. harveyi* and *S. agalactiae* [12], while *Bacillus subtilis* and *Lactobacillus plantarum* exert antagonistic effects on *S. agalactiae* [13]. In the current research, we observed the antagonistic ability of *Bacillus* to be more prominent, particularly that of *Bacillus cereus* (*B. cereus*), which is a gram-positive bacterium. Moreover, while the potential of *B. cereus* for antagonism on *S. agalactiae* and Vibrio splendidus has been reported [14, 15], the basis of its antibacterial mechanism on these three aquatic pathogens is unclear.

*B. cereus* could secrete a variety of substances in the metabolic process, including polyketide biosynthesis, sugars, proteases, peptides, bacteriocins, and other metabolites [16]. Investigation of the antimicrobial substances of *B. cereus* has mainly focused on peptides synthesized by non-ribosomal pathways and proteins synthesized by ribosomal pathways [17, 18]. The liquid chromatography-tandem high-resolution mass spectrometry (LC-MS) technique is often used to separate and identify antimicrobial substances. *Bacillus malacitensis* Z-5 antagonized *Verticillium dahlia* Kleb, and 8 lipopeptides were identified by LC-MS, including C_14_ surfactin B, C_14_–C_17_ iturin A, and C_14_, C_15_, and C_17_ iturin B [19]. In addition, Kannurin, a cyclic lipopeptide belonging to the surfactin class, was identified in *Bacillus cereus* AK1 by LC-MS. This novel lipopeptide exhibits broad-spectrum activity against clinically relevant yeasts and molds [17] and was measured in the antibacterial spectrum of *Bacillus cereus* BA09. LC-MS identified the antibacterial peptide substance has a closed, circular structure and contains the three amino acids Leu, Gln, and His [18]. Whilst one-dimensional liquid chromatography (1D-LC) is limited in the face of complex material systems [20], two-dimensional liquid chromatography (2D-LC) is a technique based on 1D-LC in which the fraction is subjected to further separation after eluting from the one-dimensional (1D) column. It enables interfering substances to be eliminated from complex samples so that required fractions can be selectively analyzed. Not only does 2D-LC improve the resolution and peak capacity of the chromatographic system, it also improves the selectivity and sensitivity of tandem mass spectrometry [21]. At present, there is no record of using 2D-LC-MS/MS to separate and identify the antimicrobial substances of *B. cereus*.

In this study, the screening and identification of antagonistic bacteria against *E. tarda*, *V. Harvey*, and *S. agalactis* were simultaneously conducted. The antibacterial substances were extracted by methanol, then separated and identified through 2D-LC-MS/MS. The results provide a basic interpretation of the antibacterial substance of *B. cereus* and serve as a useful reference for the research and development of aquaculture drugs.

## Materials and Methods

### Strain Source and Culture

The aquatic pathogen indicator bacteria used in this study were *E. tarda*, *V. harveyi*, and *S. agalactiae*, all cultured under the conditions of 180 r/min at 28°C in nutrient broth (NB) medium with a salinity of 30‰ for 12 h. Fourteen strains of *Lactobacillus* were grown by inverted culture in de Man, Rogosa and Sharpe (MRS) agar with a salinity of 10‰ at 28°C for 24 h. Finally, 5 strains of *Bacillus* were also cultured upside down in nutrient agar (NA) medium with a salinity of 30‰ at 28°C for 24 h. These strains were selected for screening as they displayed broad-spectrum antagonistic effects in previous screening work. *Lactobacillus* are labeled with LAB+ number, and *Bacillus* are labeled with BA+ number. The above strains were all obtained from the Aquatic Animal Hospital of Dalian Ocean University. The media used were all purchased from Haibo Biotechnology Co., Ltd. in Qingdao Hi-Tech Industrial Park.

### Antagonistic Bacterial Screening

The preliminary screening of antagonistic bacteria was implemented through the double-plate method [22]. Semi-solid medium (7‰ agar, NB with 30‰ salinity) containing 10^6^ CFU/ml of each pathogenic indicator bacteria was poured onto the solid medium of the screening strains, which had been dibbling cultured for 6 h. After culturing at 28°C for 12 h (*V. harveyi* and *S. agalactiae*) or 24 h (*E. tarda*), the bacteriostatic circle diameters (BCDs) were recorded. The antagonistic rescreening was carried out through the punching method [23]. The strains obtained during the preliminary screening were cultured in a liquid medium for 6 h, followed by a secondary culture with a 1% inoculation amount at 28°C and 180 r/min for 36 h. The fermentation liquor of the BA09 strain was centrifuged at 4,000 ×*g* for 10 min at 4°C. The supernatant was filtered by a 0.22 μm filter membrane to obtain sterile fermentation broth. A 5-mm puncher was used to punch holes in a semi-solid medium containing pathogenic indicator bacteria, to which 70 μl of sterile fermentation broth was added. After culturing at 28°C for 12 h (*V. harveyi* and *S. agalactiae*) or 24 h (*E. tarda*), the BCDs were recorded.

### Identification of Antagonistic Strain BA09

The strain with the most antagonistic effect was subjected to DNA extraction. The 16S rDNA universal primers 27F (5'-AGAGTTTGATCCTGGCTCAG-3') and 1492R (5'-GGTTACCTTGTTACGACTT-3') were used. Each 25-μl PCR reaction system contained 1 μl of template DNA, 2.5 μl of 10× buffer (Mg^2+^), 1 μl of dNTP, 0.25 μl of Taq DNA polymerase, 2 μl of primer mix, and 18.25 μl of double-distilled water. The PCR procedure was 94°C, 4 min; 30 cycles (94°C, 30 s; 55°C, 30 s; 72°C, 90 s); and 72°C, 10 min. The sequencing of PCR products was implemented at Beijing Huada Gene Technology Co. The sequencing results were submitted to NCBI for GenBank registration and BLAST comparison. The taxonomic status of strain BA09 was determined using the neighbor-joining method within the MEGA 11 program to construct the phylogenetic tree. The physiological and biochemical indicators were determined utilizing biochemical identification tubes (Binhe Microorganism Reagent Co., Ltd., China).

### Extraction of Bacteriostatic Substances and Activity Determination

Two hundred milliliters of sterile fermentation broth was vacuum lyophilized in a freeze dryer at -65°C and weighed. The obtained lyophilizate was dissolved in 200 ml of methanol and stood overnight at 4°C before being centrifuged at 6,000 ×*g* for 10 min. Following that, the obtained supernatant was concentrated using rotary evaporation at 40°C under a vacuum, and was then redissolved in 200 ml of water, including the above methanol-insoluble precipitate. Finally, the bacteriostatic activities were confirmed by using the punching method as described above.

### 1D Preparation of Methanol Extract of Strain BA09

The methanol extract of strain BA09 was desalted in pure water using a 1000 Da dialysis bag. It was then lyophilized, weighed, and redissolved in water to 100 mg/ml. The bacteriostatic activity was confirmed by using the punching method. The 1D-LC preparation was performed on an Alliance HPLC system consisting of a 2489 photodiode array detector (Waters, USA). The column used was the XCharge C_18_ (10 × 250 mm, 10 μm, Acchrom, China). The mobile phase A was ACN, and B was 0.1% FA (v/v) in water. The gradient program was 0 min (5% A) - 25 min (45% A) - 44 min (55% A) - 45 min (85% A) - 55 min (95% A), with elution at a flow rate of 3.3 ml/min. Chromatograms were recorded at 210 nm and 230 nm. The injection volume was 100 μl, and a total of 20 injections were implemented. A total of 27 fractions, named F1-F27, were collected every 2 min, from 2 min to 56 min. The total mass of the fractions after nitrogen blow-drying was 162.1 mg. After merging each injection, the fractions were dried under nitrogen and redissolved in water at a concentration of 10 mg/ml. The bacteriostatic activities of each fraction were confirmed by using the punching method.

### 1D-LC Analysis and 2D-LC Separation of Active Fractions

The 6 fractions with bacteriostatic activities, F16, F17, F19, F20, F21, and F26, were subjected to 1D-LC analysis and 2D-LC separation, with the former being performed on the XCharge C_18_ column (4.6 × 250 mm, 7 μm, Acchrom). The mobile phase A was ACN, and B was 0.1% FA (v/v) in water. The gradient program was 0 min (5% A) - 25 min (45% A) - 44 min (55% A) - 45 min (85% A) - 55 min (95% A), with elution at a flow rate of 0.7 ml/min. The injection volume was 10 μl. Chromatograms were recorded at 230 nm. The 2D-LC separation was performed on the C_18_ME column (4.6 × 150 mm, 5 μm, Acchrom, China). The mobile phase A was ACN, and B was 0.1%TFA (v/v) in water. The gradient program was 0 min (45% A) - 60 min (95% A), with elution at a flow rate of 0.7 ml/min. The injection volume was 5 μl. Chromatograms were recorded at 230 nm.

### 2D-LC-MS/MS Analysis of Active Fractions

The 2D-LC-MS/MS analysis of active fractions was performed on an Agilent 1290 Infinity ultra-high performance liquid chromatography (UHPLC) system coupled with an Agilent 6545 Quadrupole-Time of Flight (Q-TOF) mass spectrometer (Agilent, USA) equipped with an ESI source. The column used was a C_18_ME (2.1 × 150 mm, 5 μm, Acchrom, China). The flow rate was 0.2 ml/min and the injection volume was 5 μl. Other chromatographic conditions mirrored those of 2D-LC separation. The mass spectrometer conditions were as follows: nebulizer gas pressure (35 psi), drying gas flow rate (8 L/min), gas temperature (350°C), capillary voltage (3,000 V), fragmentor voltage (75 V) and collision energy (35 eV). The MS scan ranged from 100 to 1,000 m/z. MS was scanned from 100 to 4,000 m/z and MS/MS was scanned from 50 to 4,000 m/z. The analysis was operated in a positive mode.

## Results

### Screening of Antagonistic Strains

The bacteriostatic activity screening of 19 strains against *E. tarda*, *V. harveyi*, and *S. agalactiae* was performed first. The results showed that 11 strains of *Lactobacillus* had different degrees of antagonistic effects on the three pathogens, of which LAB8, LAB16, and LAB18 had the strongest antagonistic effects on *E. tarda*, *V. harveyi*, and *S. agalactiae*, respectively. Among the *Bacillus*, only BA09 and BA045 had antagonistic effects on the three pathogens (Table S1). Therefore, the strains LAB8, LAB16, LAB18, BA09, and BA045 were subjected to re-screening, as shown in Table 1. Except for LAB8, the remaining 4 strains all had inhibitory effects on the three pathogens, among which BA09 showed the best inhibitory effects, and was therefore subjected to further study.

### Identification of Strain BA09

**Fig. 1:**
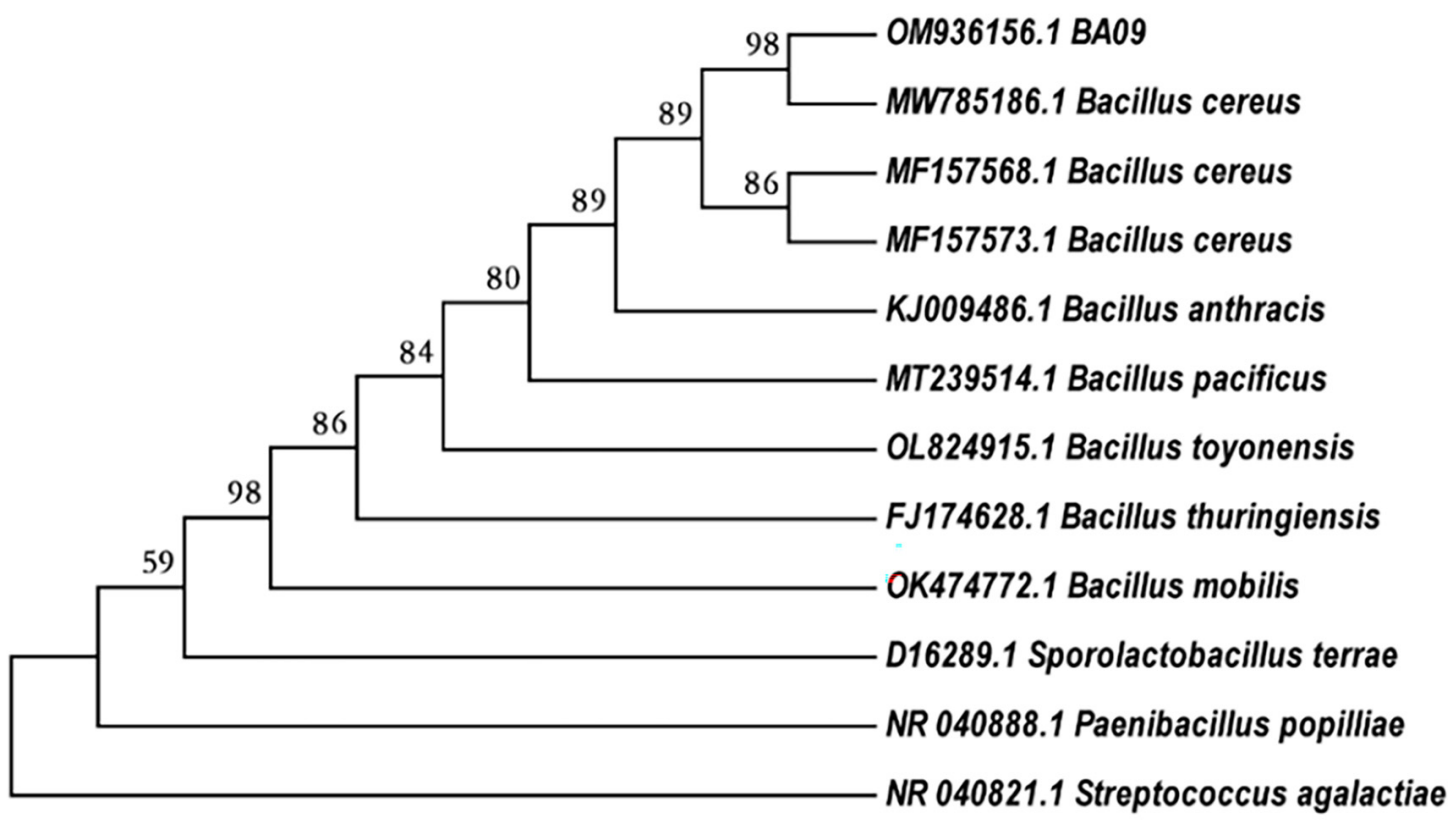
Phylogenetic tree of strain BA09.

The sequencing results for strain BA09 were submitted to GenBank (Accession No. OM936156.1), and based on the BLAST results, the strain showed 99.08% identity with *Bacillus cereus* (MN309968.1). Moreover, BA09 clustered with *Bacillus cereus* (MW785186.1) in the phylogenetic tree (Fig. 1). The physiological and biochemical results were consistent with the reference strain *Bacillus cereus* ATCC 14579 (Table S2). Therefore, strain BA09 was identified as *Bacillus cereus*.

### Extraction of Antibacterial Substances and Activity Determination

**Fig. 2:**
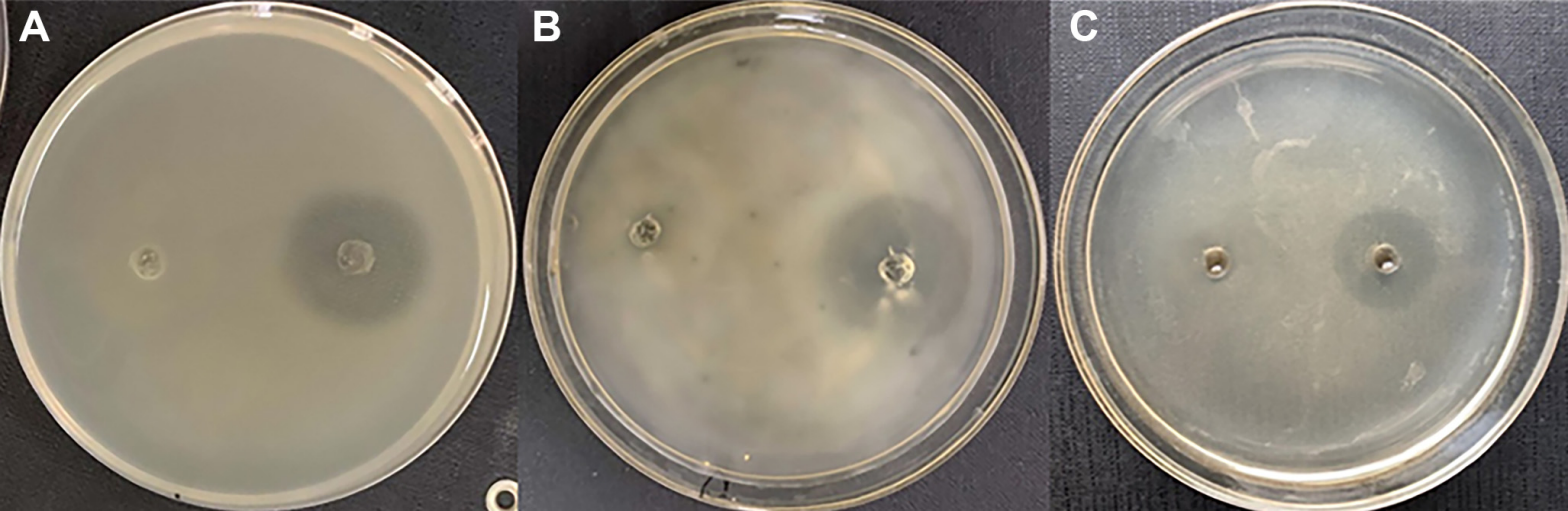
The bacteriostatic circle of the methanol extract (the left hole of each plate) and the methanolinsoluble part (the right hole of each plate) on *E. tarda* (A) *V. harveyi* (B) *S. agalactiae* (C).

After being lyophilized, the sterile fermentation broth of strain BA09 reached a mass of 4.15 g, and following extraction with methanol, 1.51 g of crude extract was obtained. The bacteriostatic activities of the methanol-soluble and methanol-insoluble parts were tested. As shown in Fig. 2, the methanol extract displayed inhibition zones with different diameters against the three pathogens, while the methanol-insoluble substances did not produce BCDs.

### 2D-LC Separation of Bacteriostatic Ingredients

**1D-RPLC Preparation and Antibacterial Activity Tracking**

**Fig. 3:**
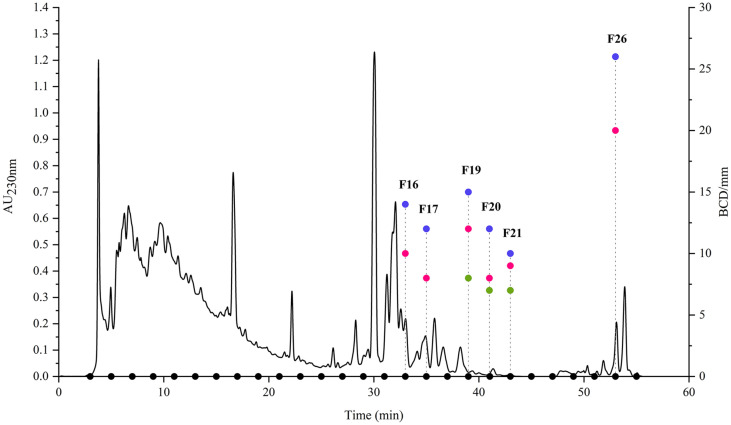
The 1D-RPLC preparation chromatography (230 nm) of methanol extract from strain BA09 and BCD of 27 1D fractions (collected every 2 min) on *E. tarda*, *V. harveyi*, *S. agalactiae*. Purple dots: BCD on *V. harveyi*; pink dots: BCD on *S. agalactiae*; green dots: BCD on *E. tarda*; black dots: 27 1D fractions.

The methanol extract of strain BA09 retained antibacterial activity after desalination, and was subjected to 1D-RPLC preparation to track the bacteriostatic ingredients. Through repeated preparation, a total of 27 fractions, named F1-F27, were time-based collected. After drying, the BCDs against the three aquatic pathogens were measured for these fractions. As shown in Fig. 3, six fractions displayed antibacterial activities, including F16, F17, F19-F21, and F26. Among these, F19-F21 had inhibitory activities against all three pathogens, with BCDs of 8, 15, and 12 mm (F19), 7, 12, and 18 mm (F20), and 7, 10, and 9 mm (F21) against *E. tarda*, *V. harveyi*, and *S. agalactiae* respectively. The other three fractions F16, F17, and F26 only have antibacterial activities against *V. harveyi* and *S. agalactiae*, showing BCDs of 14 and 10 mm (F16), 12 and 8 mm (F17), and 26 and 20 mm (F26) against *V. harveyi* and *S. agalactiae*, respectively. Except for these 6 fractions, no other fractions had antibacterial activities.

### 2D-LC Separation of Active Fractions

**Fig. 4:**
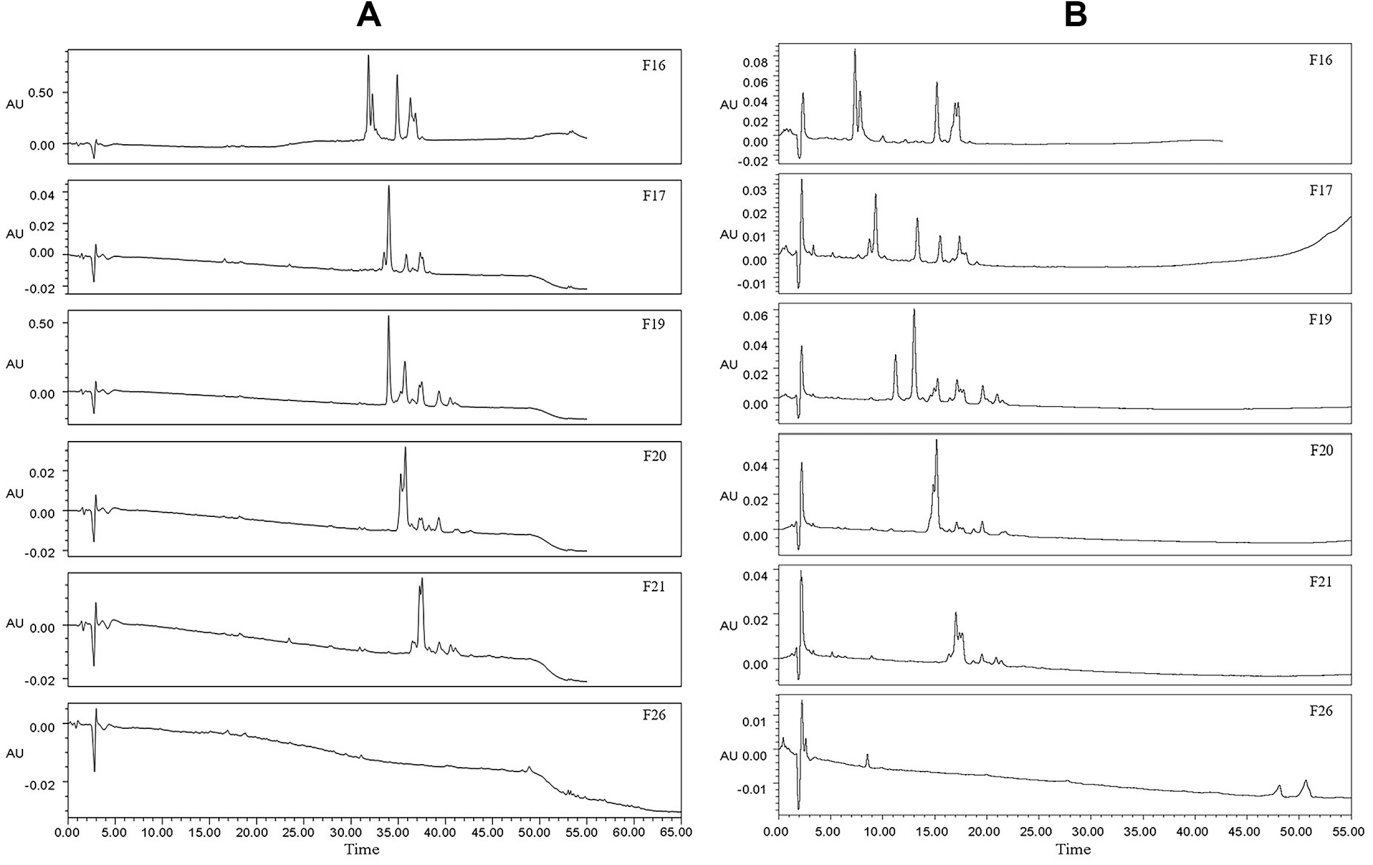
Chromatographic analysis of 6 active fractions on 1D column XCharge C_18_ (A) and 2D column C_18_ME (B).

Even though the 1D-LC preparation was performed, the fractions obtained were still not monomeric, which made it difficult to identify bacteriostatic ingredients. Fig. 4A shows the chromatograms of the 6 active fractions analyzed using the same conditions as preparation, in which chromatographic peaks were particularly concentrated and distributed. Considering the resolution requirement of tandem mass spectrometry, the 2D-LC separation implemented on another C_18_ME column was performed (Fig. 4B). The chromatographic peaks are shown to be more dispersed, and more compounds were separated, such as with F17. This was due to the difference in the separation mechanism between the 1D-LC and 2D-LC columns, which was beneficial for tandem mass detection.

### LC-MS/MS Analysis of Active Fractions

**Mass Peak Detection of Active Fractions**

**Fig. 5:**
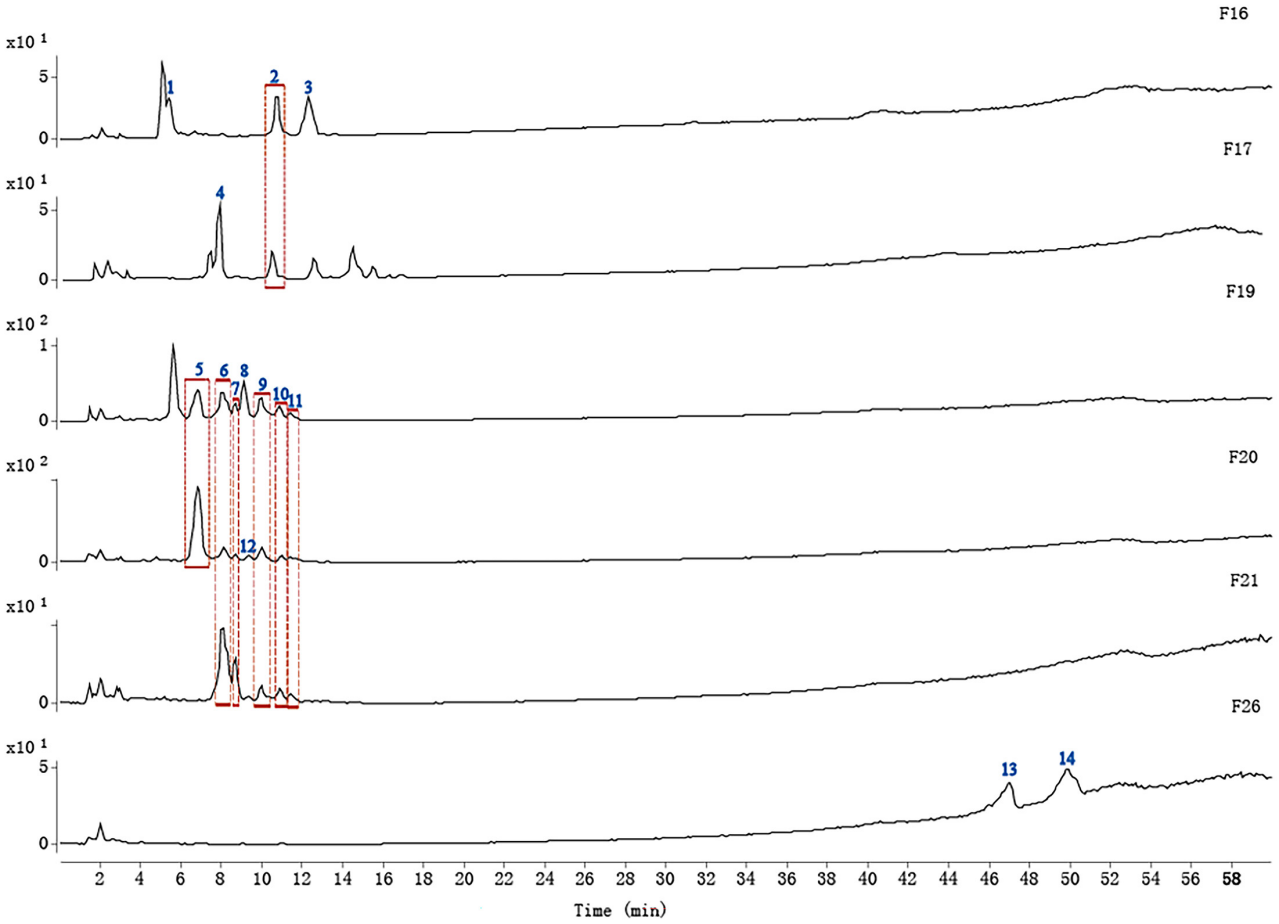
The total ion chromatograms of the six active fractions through RPLC-Q-TOF MS.

The 2D-LC separation system, coupled with Q-TOF MS/MS to characterize the chemical constituents in strain BA09, enabled us to obtain accurate molecular weights, molecular formula, and fragmentation information for each peak. The mass spectrum data were collected in positive mode, generating total ion chromatograms for the 6 active fractions (Fig. 5). Due to the existence of crossing in the process of 1D-LC preparation, identical components may enter into adjacent fractions. Therefore, the 2D-LC chromatographic peaks with the same retention time, accurate molecular weight, and fragmentation information in the adjacent fractions were identified as identical components, and also more likely to be the bacteriostatic active components. The characterization results were listed in Table 2. Among these, Cpd. 2, 5, 6, 7, 9, 10, and 11 are common components of multiple fractions. Cpd. 2 is a common component of F16 and F17, with the same retention time of 10 min, the accurate molecular weight of 1463.80, with fragment ions, and was identified as C_16_ fengycin A. Similarly, Cpd. 5 is a common component of F19 and F20 and was identified as C_16_ fengycin B. Cpd. 6, 7, 9, 11 are common components of F19, F20, and F21 and were identified as C_17_ fengycin B, C_15_ fengycin A uns, C_15_ fengycin B uns, and C_16_ fengycin B uns, respectively. Meanwhile, Cpd. 10 is a common component of F19 and F21 and was identified as C_18_ fengycin B. In addition to these common components, Cpd. 1 and 3 in F16 were identified as C_15_ bacillomycin D and C_17_ fengycin A; Cpd. 4 in F17, Cpd. 8 in F19, and Cpd. 12 in F20 were identified as C_16_ bacillomycin D, C_17_ bacillomycin D, and C_18_ fengycin A, respectively. Cpd. 13 and 14 in F26 were identified as C_14_ surfactin A/C and C_15_ surfactin A/C. All identified components are lipopeptides.

### Characterization of Lipopeptides in *Bacillus cereus*

**Fig. 6:**
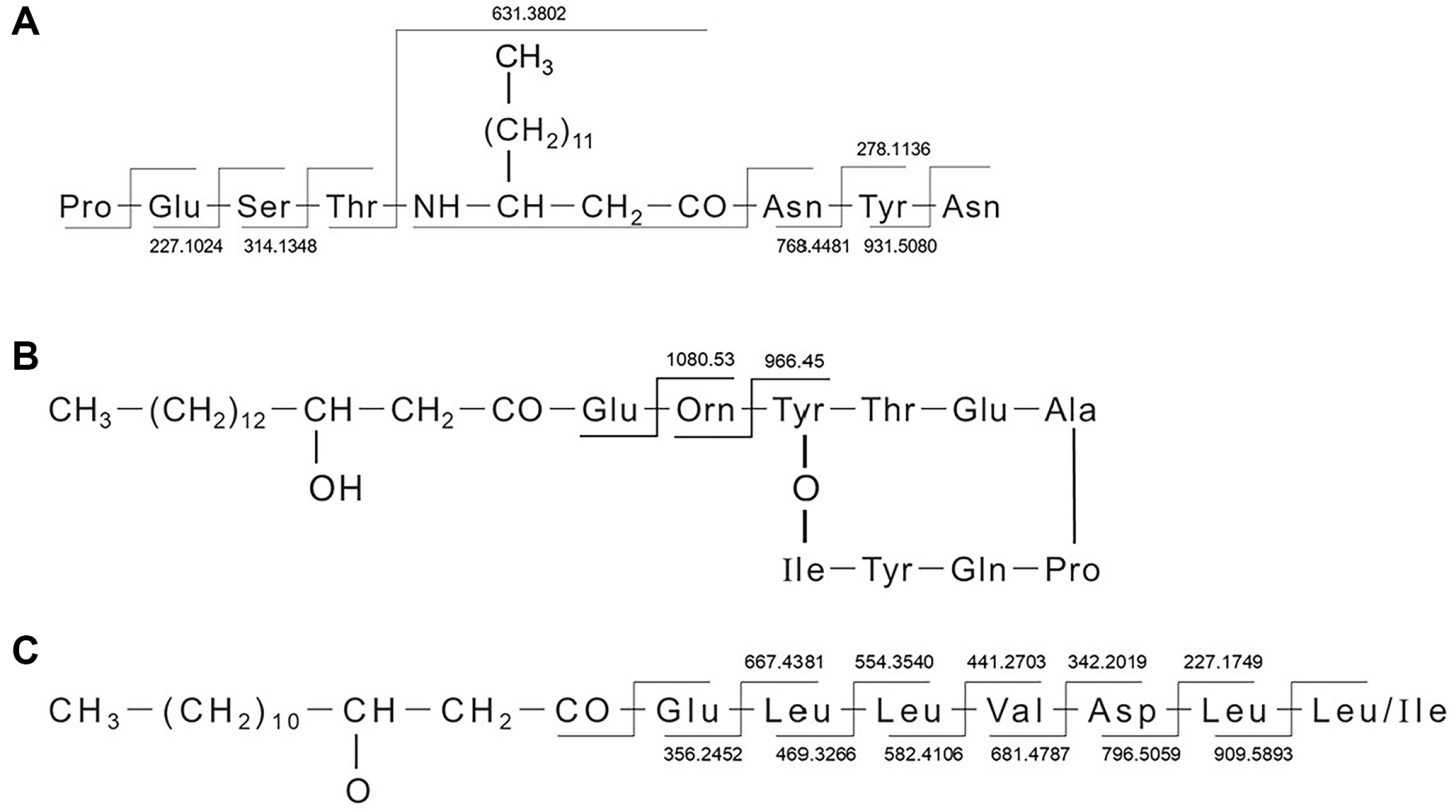
Structure fragmentation diagram of Cpd. 1 (A) Cpd. 2 (B) Cpd.13 (C).

Based on the accurate m/z values of parent ions and fragment ions, as well as the fragmentation pattern reported in the literature, a total of 3 types of lipopeptides were characterized, including fengycin, bacillomycin, and surfactin. Cpd 1, 2 and 13 were taken as examples to interpret the fragmentation patterns of each lipopeptide type. Cpd 1, 2 and 13 showed [M+H] ^+^ ions at m/z 1045.5582, 1463.803,1022.677, which gave rise to a molecular formula of C_49_H_76_N_10_O_15_, C_72_H_110_N_12_O_20_, C_52_H_91_N_7_O_13_ (Table 2). Additionally, Cpd 1, 2 and 13 belonged to bacillomycin, fengycin, and surfactin, respectively. The ion peaks of each molecule were further collided and induced dissociation, resulting in characteristic ion fragments of different types. The number of carbon atoms on the fatty acid chain was deduced by calculating the difference between the molecular weight and the molecular weight of the peptide chain. For example, Cpd 1 showed [M+H] ^+^ ions at m/z 1045.5582, and the molecular weight of amino acids in the peptide chain is 97.05227, 129.0426, 87.03203, 101.04768, 114.04293, 163.06333, and 114.04293. The difference between the m/z and peptide chain is about 240, which subtracts the molecular weight of the fixed part of the fatty acid group 85. Cpd. 1 was identified as C_15_ bacillomycin D (the amino acid sequence of bacillomycin peptide chain is Asn-Tyr-Asn-Pro-Glu-Ser-Thr [27]) (Fig. 6A). The difference between the m/z of Cpd 2 and the molecular weight of the peptide chain is about 254, which subtracts the molecular weight of the fixed part of the fatty acid group 87, obtaining the difference of 167, which is divided by 14, obtaining 12 of other carbons. Cpd. 2 was identified as C_16_ fengycin A (the amino acid sequence of the fengycin peptide chain is Glu-Orn-Tyr-Thr-Glu-Ala (Val) -Pro-Gln-Tyr-Ile [25]) (Fig. 6B). The difference between the m/z of Cpd. 13 and the molecular weight of the peptide chain is about 226, which subtracts the molecular weight of the fixed part of the fatty acid group 86, obtaining the difference of 140, which is divided by 14, obtaining 10 of other carbons. Cpd. 13 was identified as C_14_ surfactin A/C (the amino acid sequence of surfactin peptide chain is Glu-Leu-Leu-Val-Asp-Leu-Leu-Leu [32]) (Fig. 6C).

## Discussion

The strain BA09 screened in this study showed antagonistic effects against *E. tarda*, *V. harveyi*, and *S. agalactiae* at the same time, and was identified as *Bacillus cereus*, which is reported to be a species with broad-spectrum antagonism [14, 15]. However, the research on the inhibitory substances in *B. cereus* against these three pathogens is not extensive. Thus, it is necessary to identify the bacteriostatic components of *B. cereus*, along with their material basis and mechanism.

LC-MS is usually used to profile and identify microbial metabolites [17-19]. The separation ability of LC directly affects the resolution of tandem mass detection. Using 1D-LC-MS, H. Shabeer Ali and Zhang Lina identified 5 and 1 lipopeptides in *B. cereus*, respectively [17, 18]. In this study, 2D-LC-MS was adopted to analyze and identify the bacteriostatic components in *B. cereus*. The methanol extract of strain BA09 was first separated and prepared by 1D-RPLC. Six 6 active fractions were then located through antibacterial activity screening, and subjected to 2D-RPLC-MS/MS for component identification. A total of 14 lipopeptides were identified, which was greater than the number identified by 1D-LC-MS, suggesting the excellent separation and identification efficiencies of 2D-LC-MS.

A total of 14 lipopeptides were identified in the active fractions of strain BA09, which simultaneously had antagonistic effects on *E. tarda*, *V. harveyi*, and *S. agalactiae*. Lipopeptides are widely present in *Bacillus* and have a variety of structure types. They are non-specific, antibacterial, and do not easily produce drug resistance in host cells, whose mechanism is different from the antibiotics [34, 35]. The 14 identified lipopeptides belong to 3 types, including fengycin, bacillomycin, and surfactin, which are also the main antibacterial lipopeptides. Unlike bacillomycin and surfactin, fengycin lipopeptides produce only 2 characteristic ion fragments in the process of fragmentation, which distinguished them from the other two types of lipopeptides and showed obvious regularity. Due to the partially looping peptide chain, the amino acids are not easily broken. When the sixth position of the fengycin peptide chain is Ala, it is fengycin A; and if the sixth position is Val, it is fengycin B [36]. Fengycin A produces typical fragments of m/z 1080 and 966, and fengycin B produces typical fragments of m/z 1108 and 994 [36]. Compared with fengycin, bacillomycin and surfactin obtained more characteristic ion fragments, and presumably because the peptide chain is partially straight, the amino acids are prone to breakage. There are four common characteristic ion fragments?of?m/z?342.201, 441.270, 554.354, and 667.438 produced?by surfactin, while there are no characteristic ion fragments produced?by?bacillomycin. This provides a reference for the identification of lipopeptides through their fragmentation characteristics.

In this study, nine lipopeptides were considered the most likely antibacterial substances for *B. cereus* BA09. Cpd. 6, 7, 9 and 11 (C_17_ fengycin B, C_15_ fengycin A uns, C_15_ fengycin B uns, C_16_ fengycin B uns) were identified as the common components of F19, F20, and F21, which were found to exert antibacterial effects on the three pathogens. Therefore, it is speculated that these 4 lipopeptides are the main components of strain BA09 responsible for antibacterial effects. Similarly, Cpd. 5, 10 (C_16_ fengycin B, C_18_ fengycin B) simultaneously existed in F19, F20, F19, and F21, respectively, also indicating the major roles in the antibacterial effects of these 2 lipopeptides. F16, F17, and F26 only had antibacterial effects on *V. harveyi* and *S. agalactiae*. Cpd. 2 (C_16_ fengycin A) was the common component of F16 and F17, suggesting the possibility of antibacterial effects on *V. harveyi* and *S. agalactiae*. F26 had no crossover with other active fractions, in which Cpd. 13 and Cpd. 14 (C_14_ surfactin A/C, C_15_ surfactin A/C) were identified. Thus, they were presumed to be antibacterial lipopeptides against *V. harveyi* and *S. agalactiae*. Overall, the main antibacterial lipopeptides mostly belong to fengycin, which plays a leading role in the antibacterial process of *B. cereus*. The fengycins identified in this study are structurally consistent with previous reports, but it is worth noting that unlike their sources, the antibacterial potential exhibited is also different. This study shows that the fengycin isolated in BA09 can inhibit three pathogens, and whether this powerful antibacterial potential is related to the source is worthy of further study.

Fengycin is categorized into fengycin A and B, which mainly display antagonistic effects against fungi and have limited inhibitory effects on bacteria [37]. However, in this study, fengycin was found to be the major antibacterial substance against three aquatic pathogens. This result is consistent with Yanjun Gús [38] work, in which C_17_ fengycin B, identified in strains of *Bacillus* spp., was inferred to be antagonistic against *Staphylococcus aureus*, *Listeria monocytogenes*, *Salmonella* and *Pseudomonas aeruginosa*. In addition, the antagonistic mechanism of fengycin is reportedly involved in the accumulation of reactive oxygen species and cell membrane damage in both fungi and bacteria [38, 39]. Currently, the antagonistic mechanism of lipopeptides on *E. tarda*, *V. harveyi*, and *S. agalactiae* is unclear and will be more rigorously studied in the future.

## Supplemental Materials

Supplementary data for this paper are available on-line only at http://jmb.or.kr.



## Figures and Tables

**Table 1:** BCDs of five strains against three pathogens (mm).

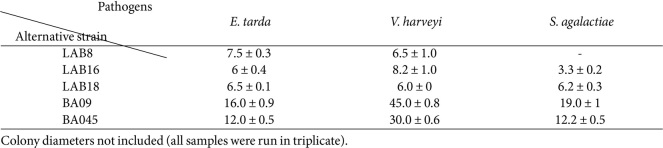

**Table 2:** Tentatively identified lipopeptides in *Bacillus cereus*.

No.	Fra.	t _R_/min	m/z	Measured ions	Formula	Fragment ions	Fragment ions	Tentative Identification
1	F16	5.39	1045.5582	[C_49_H_77_N_10_O_15_] ^+^	C_49_H_76_N_10_O_15_	227.1024, 278.1136, 314.1348, 631.3802, 768.4481, 931.5080	C_15_ bacillomycin D	[24]
2	F16	10.799	1463.803	[C_72_H_111_N_12_O_20_] ^+^	C_72_H_110_N_12_O_20_	966.4538, 1080.5336	C_16_ fengycin A	[25]
	F17	10.599	1463.8071			966.4529, 1080.5308		
3	F16	12.301	1477.819	[C_73_H_113_N_12_O_20_] ^+^	C_73_H_112_N_12_O_20_	966.4548, 1080.5306	C_17_ fengycin A	[26]
4	F17	7.197	1059.5738	[C_50_H_79_N_10_O_15_] ^+^	C_50_H_78_N_10_O_15_	115.0865, 227.1022, 278.1131, 314.1342, 392.1556, 415.1838, 645.3954, 668.4171, 746.4426, 782.4642, 833.4753, 945.5255	C_16_ bacillomycin D	[27]
5	F19	6.795	1491.8391	[C_74_H_115_N_12_O_20_] ^+^	C_74_H_114_N_12_O_20_	994.4862, 1108.5610	C_16_ fengycin B	[25]
	F20	6.810	1491.8401			994.4869, 1108.5658		
6	F19	8.097	1505.8560	[C_75_H_117_N_12_O_20_] ^+^	C_75_H_116_N_12_O_20_	994.4847, 1108.5664	C_17_ fengycin B	[28]
	F20	8.112	1505.8508			994.4835, 1108.5536		
	F21	8.100	1505.8545			994.4831, 1108.5648		
7	F19	8.698	1447.8123	[C_71_H_107_N_12_O_20_] ^+^	C_71_H_106_N_12_O_20_	966.4538, 1080.5370	C_15_ fengycin A uns	[29]
	F20	8.713	1447.8081			966.4531, 1080.5328		
	F21	8.701	1447.8126			966.4554, 1080.5282		
8	F19	9.098	1073.5898	[C_51_H_81_N_10_O_15_] ^+^	C_51_H_80_N_10_O_15_	115.0860, 227.1023, 278.1134, 314.1341, 392.1559, 415.1828, 659.4116, 682.4360, 760.4576, 796.4791, 959.5415, 976.5293	C_17_ bacillomycin D	[30]
9	F19	10.000	1475.8443	[C_73_H_111_N_12_O_20_] ^+^	C_73_H_110_N_12_O_20_	994.4853, 1108.5665	C_15_ fengycin B uns	[29]
	F20	10.014	1475.8416			994.4830, 1108.5704		
	F21	10.003	1475.8395					
10	F19	10.902	1519.8681	[C_76_H_119_N_12_O_20_] ^+^	C_76_H_118_N_12_O_20_	994.4868, 1108.5612	C_18_ fengycin B	[31]
	F21	10.905	1519.8655					
11	F19	11.403	1489.8549	[C_74_H_113_N_12_O_20_] ^+^	C_74_H_112_N_12_O_20_	994.4894, 1108.5618	C_16_ fengycin B uns	[29]
	F20	11.417	1489.8554					
	F21	11.406	1489.8538					
12	F20	9.313	1491.8338	[C_74_H_115_N_12_O_20_] ^+^	C_74_H_114_N_12_O_20_	966.4506, 1080.5211	C_18_ fengycin A	[25]
13	F26	46.952	1022.6771	[C_52_H_92_N_7_O_13_] ^+^	C_52_H_91_N_7_O_13_	227.1749, 342.2019, 356.2452, 441.2703, 469.3266, 554.3540, 582.4106, 667.4381, 681.4787, 796.5059, 909.5893	C_14_ surfactin A/C	[32]
14	F26	49.956	1036.693	[C_53_H_94_N_7_O_13_] ^+^	C_53_H_93_N_7_O_13_	227.1750, 342.2018, 370.2583, 441.2706, 483.3424, 554.3542, 596.4263, 667.4380, 695.4948, 810.5226, 923.6060	C_15_ surfactin A/C	

‘uns’ means the containing of the unsaturated bond.
