# Supplementary material for: Screening of Antagonistic Bacteria against Three Aquatic Pathogens and Characterization of Lipopeptides in Bacillus cereus BA09
Source: J Microbiol Biotechnol. 2024 Aug 25;34(10):2023–32. doi: 10.4014/jmb.2404.04017 (PMC11540599; doi:10.4014/jmb.2404.04017)
Supplement: Supplementary file 1 [file jmb-34-10-2023-supple.pdf]

J. Microbiol. Biotechnol. 2024. 34(10): 2023–2032
https://doi.org/10.4014/jmb.2404.04017

Table S1. Preliminary screening results.

Pathogenic strain / Alternative strain	*Edwardsiella tarda*	*Vibrio harveyi*	*Streptococcus agalactiae*
LAB1	++	++	++
LAB2	++	+	+
LAB3	++	++	+
LAB4	++	++	-
LAB5	++	+	++
LAB7	++	++	++
LAB8	+++	+	-
LAB9	++	+++	+
LAB11	+	+++	-
LAB13	+	+++	+
LAB15	++	++	+
LAB16	++	++++	+
LAB18	+	++	+++
LAB19	+	++	+
BA09	++	+	++
BA045	++	+	++
BA90	-	+	+
BA91	++	-	-
BA92	-	+	-

Notes: The data is the difference of the diameter between the inhibition circle and the colony.
+：d<3mm； ++：3mm≤d<6mm； +++：6mm≤d<9mm； ++++：d≥9mm； -：non-effective

Table S2. The physiological-biochemical characteristic of the antagonistic strain BA09.

Characteristics	*Bacillus cereus* ATCC 14579 [1]	BA09
Mannitol	-	-
Hydrolyzes starch	+	+
V. P.	+	+
Nitrate reduction	+	+
β-galactosidase	-	-
Citrate	+	+
Gelatin	+	+
Glycerol	-	-
Ribose	+	+
Galactose	-	-
D-Mannose	-	-
Arbutin	+	+
Salicin	+	+
Cellobiose	+	+
Sucrose	+	+
Trehalose	+	+
Amylum	+	+
Glycogen	+	+

Notes：+. positive, −. negative

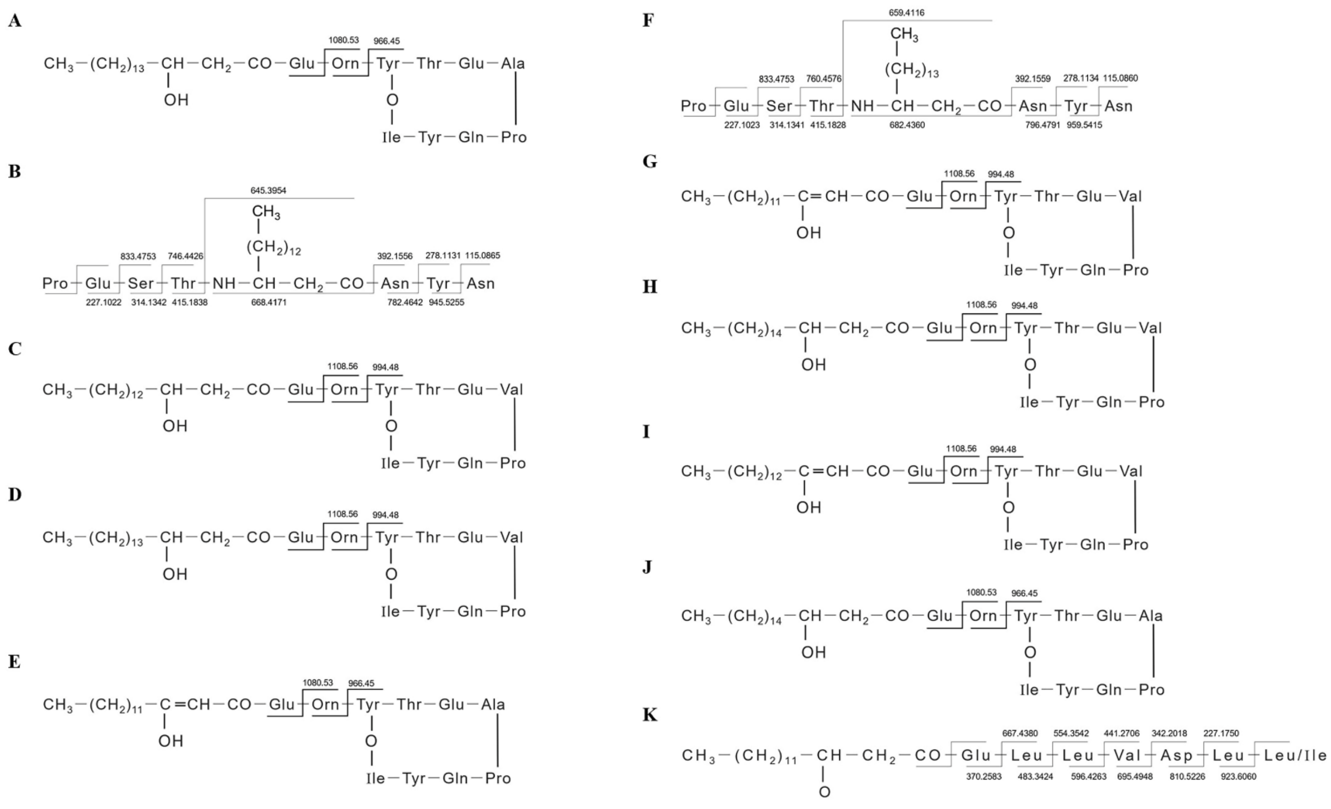

Fig. S1. Structure fragmentation diagrams of Cpd. 3 (A) Cpd. 4 (B) **Cpd.5** (C) **Cpd. 6** (D) **Cpd. 7** (E) **Cpd.8** (F) **Cpd. 9** (G) **Cpd. 10** (H) **Cpd.11** (I) **Cpd. 12** (J) **Cpd. 14** (K).

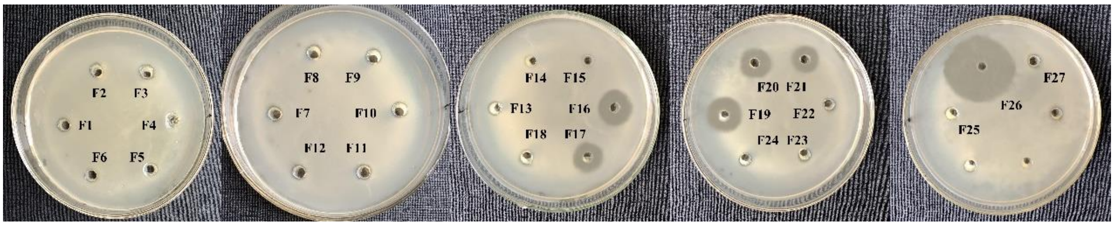

Fig. S2. Antibacterial effect of 27 fractions on *Vibrio harveyi*. (unmarked part, only punched without added substance).

[1] Guinebretière M-H, Auger S, Galleron N, Contzen M, De Sarrau B, De Buyser M-L, *et al.* 2013. *Bacillus cytotoxicus* sp. nov. is a novel thermotolerant species of the *Bacillus cereus* Group occasionally associated with food poisoning. *Int. J. Syst. Evol. Microbiol*. **63**: 31–40.
